# Progression of diabetes, heart disease, and stroke multimorbidity in middle-aged women: A 20-year cohort study

**DOI:** 10.1371/journal.pmed.1002516

**Published:** 2018-03-13

**Authors:** Xiaolin Xu, Gita D. Mishra, Annette J. Dobson, Mark Jones

**Affiliations:** The University of Queensland, School of Public Health, Centre for Longitudinal and Life Course Research, Brisbane, Australia; Scripps Translational Science Institute, UNITED STATES

## Abstract

**Background:**

The prevalence of diabetes, heart disease, and stroke multimorbidity (co-occurrence of two or three of these conditions) has increased rapidly. Little is known about how the three conditions progress from one to another sequentially through the life course. We aimed to delineate this progression in middle-aged women and to determine the roles of common risk factors in the accumulation of diabetes, heart disease, and stroke multimorbidity.

**Methods and findings:**

We used data from 13,714 women aged 45–50 years without a history of any of the three conditions. They were participants in the Australian Longitudinal Study on Women's Health (ALSWH), enrolled in 1996, and surveyed approximately every 3 years to 2016. We characterized the longitudinal progression of the three conditions and multimorbidity. We estimated the accumulation of multimorbidity over 20 years of follow-up and investigated their association with both baseline and time-varying predictors (sociodemographic factors, lifestyle factors, and other chronic conditions).

Over 20 years, 2,511 (18.3%) of the women progressed to at least one condition, of whom 1,420 (56.6%) had diabetes, 1,277 (50.9%) had heart disease, and 308 (12.3%) had stroke; 423 (16.8%) had two or three of these conditions. Over a 3-year period, the age-adjusted odds of two or more conditions was approximately twice that of developing one new condition compared to women who did not develop any new conditions. For example, the odds for developing one new condition between Surveys 7 and 8 were 2.29 (95% confidence interval [CI], 1.93–2.72), whereas the odds for developing two or more conditions was 6.51 (95% CI, 3.95–10.75). The onset of stroke was more strongly associated with the progression to the other conditions (i.e., 23.4% [95% CI, 16.3%–32.2%] of women after first onset of stroke progressed to other conditions, whereas the percentages for diabetes and heart disease were 9.9% [95% CI, 7.9%–12.4%] and 11.4% [95% CI, 9.1%–14.4%], respectively). Being separated, divorced, or widowed; being born outside Australia; having difficulty managing on their available income; being overweight or obese; having hypertension; being physically inactive; being a current smoker; and having prior chronic conditions (i.e., mental disorders, asthma, cancer, osteoporosis, and arthritis) were significantly associated with increased odds of accumulation of diabetes, heart disease, and stroke multimorbidity. The main limitations of this study were the use of self-reported data and the low number of events.

**Conclusions:**

Stroke was associated with increased risk of progression to diabetes or heart disease. Social inequality, obesity, hypertension, physical inactivity, smoking, or having other chronic conditions were also significantly associated with increased odds of accumulating multimorbidity. Our findings highlight the importance of awareness of the role of diabetes, heart disease, and stroke multimorbidity among middle-aged women for clinicians and health-promotion agencies.

## Abstract 2

Using data from the Australian Longitudinal Study on Women’s Health, Xiaolin Xu and colleagues examine the progression of diabetes, heart disease, and stroke co- and multimorbidity in middle-aged women over 20 years.

## Author summary

**Why was this study done?:**

- In an aging population, it is common for women to experience two or more of diabetes, heart disease, and stroke.
- Few published studies have investigated how women progress from a “healthy” state to having one of diabetes, heart disease, and stroke and then to multimorbidity.
- No prospective evidence is available on the roles of time-varying common risk factors (i.e., high blood pressure or obesity) in the accumulation of multimorbidity from diabetes, heart disease, and stroke—information that may be important for health promotion and risk modification.

**What did the researchers do and find?:**

- In this national prospective cohort study, 13,714 middle-aged Australian women (45–50 years old) were recruited in 1996 and have been followed for 2 decades. We collected data on their health conditions, including diabetes, heart disease, and stroke, as well as potential risk factors every 3 years until 2016.
- From early to late middle age, many more women developed a single condition than multimorbidity. However, the odds ratio for accumulation of multimorbidity (i.e., from none or one to two or three, or from two to three of diabetes, heart disease, or stroke) was much higher than the odds of developing only one new condition, compared to women who did not develop any new condition over 3 years.
- Nearly one-quarter of women who were initially diagnosed with stroke subsequently progressed to other conditions, a much higher percentage than those who were initially diagnosed with diabetes (9.9%) or heart disease (11.4%).
- Women who were obese, had hypertension, were physically inactive, were smokers, or had other chronic conditions had higher odds of accumulating diabetes, heart disease, and stroke multimorbidity over the following 3-year period than women without these characteristics.

**What do these findings mean?:**

- To our knowledge, this is the first study to delineate the accumulation of diabetes, heart disease, and stroke multimorbidity and investigate its association with both baseline and time-varying predictors in a prospective cohort study.
- These findings suggest that health promotion, interventions for modifying lifestyle factors (obesity, high blood pressure, physical inactivity, and smoking), and treatment of other chronic conditions would be potentially beneficial for preventing the accumulation of diabetes, heart disease, and stroke multimorbidity.
- For women who have had a stroke, health promotion, intervention, and treatment would be particularly important, as they appear most likely to progress to other conditions.

## Introduction

The prevalence of diabetes, heart disease, and stroke multimorbidity (co-occurrence of two or three from these conditions) has increased rapidly over the past few decades [1–3], potentially translating to excess morbidity and mortality [4,5]. As the three conditions may interact with each other and be driven by common risk factors through the life course [6,7], the relationships among them are complex.

Diabetes is a well-established risk factor for heart disease and stroke, with a stronger effect in women than in men [8,9]. Although limited, there is evidence that patients with heart disease or stroke may progress to diabetes [10]. However, much less is known about how the three conditions progress from one to another sequentially through the life course [10]. The relationship between common risk factors (e.g., high blood pressure, obesity) and individual conditions has been researched intensively [6,7], but far less is known about the roles of these risk factors in the accumulation of diabetes, heart disease, and stroke multimorbidity from a longitudinal perspective [11].

Understanding the course of diabetes, heart disease, and stroke multimorbidity and related risk factors is essential for developing medical strategies to interrupt the progression from one condition to additional conditions. Considerable evidence has already shown that early intervention with diabetes or common risk factors might have the potential to ameliorate the course or even prevent the onset of cardiovascular disease (CVD) [12–14].

The aims of this article were (a) to investigate the progression of diabetes, heart disease, and stroke multimorbidity in middle-aged women over 20 years of follow-up and (b) to determine the roles of common risk factors in the accumulation of these conditions.

## Methods

The Australian Longitudinal Study on Women’s Health (ALSWH) is an ongoing population-based cohort study that aims to investigate factors associated with health and well-being over time. The women were randomly selected from the national database of the Health Insurance Commission, the universal health insurance scheme that includes all citizens and permanent residents of Australia. Details of the study design, recruitment methods, and response rates have been described elsewhere [15,16]. The data analyses for the present study were performed following a prespecified analysis plan (S1 Text). Changes in the analysis plan were also described in S1 Text. This study is reported as per the Strengthening the Reporting of Observational Studies in Epidemiology (STROBE) guidelines (S1 Checklist).

### Ethics statement

The study was approved by the Human Research Ethics Committees of the Universities of Queensland and Newcastle. All participants signed informed consent, and all data used in the analyses were de-identified.

### Participants

This study includes data from women born between 1946 and 1951, also known as the 1946–1951 cohort in ALSWH. A total of 13,714 women aged 45–50 years responded to the first survey in 1996. Response rates to the first mailed survey (baseline) cannot be exactly specified, as some women selected for the sample may not have received the invitation (e.g., if they had died or had changed their address without notifying the Health Insurance Commission). It is estimated that 53%–56% responded to the initial invitation to participate [16]. Self-administered questionnaires were sent to the women every 3 years (apart from a 2-year interval between the first and second surveys) until 2016.

**Fig 1:**
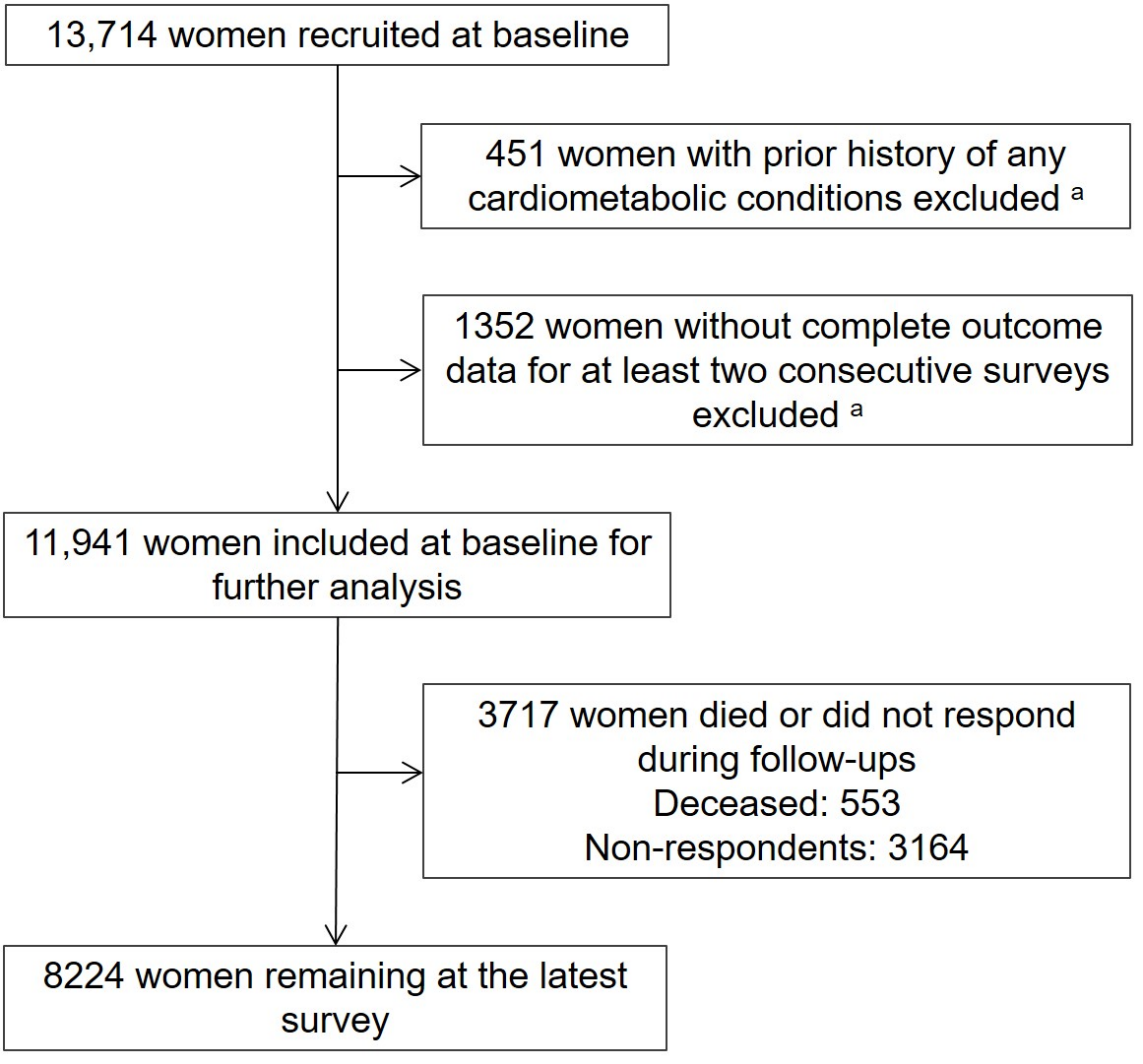
Flow diagram showing the selection of participants. ^a^Data for these women in the two boxes may overlap.

Women who participated in at least two consecutive surveys with relevant information on exposures and outcomes of interest were included in the analysis (see **Fig 1**). Attrition rates and reasons for dropout at each survey are shown in **S1 Table**.

### Outcomes

The main outcomes were the cumulative incidence of diabetes, heart disease, and stroke and accumulation of multimorbidity from these conditions. At each survey, women were asked “Have you ever been told by a doctor that you have diabetes (high blood sugar), heart disease (including heart attack, angina), and stroke over the past 3 years?” The three self-reported conditions were validated with hospital discharge data in a subset of the cohort (women living in New South Wales, Australia). The following International Statistical Classification of Diseases and Related Health Problems-Tenth Revision-Australian Modification (ICD-10-AM) diagnosis codes were used for the validation: diabetes mellitus (E10, E11, E13, and E14), ischemic heart diseases (I20–I25), and stroke (I60–I64). The prevalence and bias-adjusted kappa statistics for the three conditions were 0.93, 0.91, and 0.98, respectively [17].

The incidence of each of the conditions was based on the first report of that condition. Accumulation of multimorbidity was based on the first report of two or three of these conditions and the progression from two to three conditions.

Additionally, we decomposed multimorbidity into 4 unique outcomes that had developed over the 20-year follow-up: diabetes only, CVD only, CVD followed by diabetes, and diabetes followed by CVD [11]. CVD followed by diabetes refers to women with heart disease or stroke who subsequently developed comorbid diabetes. Diabetes followed by CVD refers to women with diabetes who subsequently developed comorbid heart disease or stroke.

### Covariates

There were four groups of covariates used in the analysis, and these covariates were collected at each survey unless indicated otherwise. Covariates included are study design variables: age in single years at 1996 (baseline), time period (Surveys 1–8); sociodemographic factors: country of birth, marital status, area of residence, education (at baseline), and ability to manage on income; lifestyle factors: body mass index (BMI), hypertension, physical activity, and smoking; and other chronic conditions: depression/anxiety, cancer, asthma, arthritis, osteoporosis, and chronic obstructive pulmonary disease (COPD). All covariates were time-varying except for country of birth and level of education.

BMI was calculated as weight in kilograms divided by height in meters squared and categorized as underweight (<18.5 kg/m^2^), normal weight (18.5–24.9 kg/m^2^), overweight (25–29.9 kg/m^2^), or obese (≥30 kg/m^2^), according to the World Health Organization classification [18]. Physical activity was categorized as sedentary (0–39 metabolic equivalent [MET] min/week), low (40–599 MET min/week), moderate (600–1,199 MET min/week), and high (≥1,200 MET min/week) [19].

### Statistical analysis

Baseline characteristics were described by the number of conditions from diabetes, heart disease, and stroke developed during the 20-year follow-up. Differences among the groups were examined using analysis of variance (ANOVA) or chi-squared tests. A proportional Venn diagram was drawn to display the number of women with single or overlapping conditions. A Sankey diagram was constructed to characterize the dynamic changes of different combinations of these conditions over time. We estimated the association among the three conditions using repeated measures logistic regression of existing conditions on the incidence of each of the other two conditions, adjusted for age at baseline and time period.

To investigate the longitudinal odds of developing these conditions, we used a repeated measures logistic regression model fitted using generalized estimating equations. We calculated odds ratios (ORs) and 95% confidence intervals (CIs) for incidence one of the three conditions or two or three over each 3-year period, compared to a reference group of women who did not develop any new conditions. We also calculated cumulative incidence of the other two conditions after the first onset of each single condition.

To estimate the associations between predictors (sociodemographic factors, lifestyle factors, and other prior chronic conditions) in the progression of diabetes, heart disease, and stroke multimorbidity, we used a generalized linear mixed model for the multinomial outcome of cumulative incidence of 0, 1, or ≥2 conditions (≥2, including the transition from 2 to 3). We calculated ORs with 95% CIs for the association between the three outcomes at each survey and the risk factors (including time-varying covariates except for education and country of birth) at the previous survey; women with 0 conditions were the reference group. For example, the cumulative incidence of 1 or ≥2 conditions from Surveys 6–7 were modelled using predictors from Survey 6. We also used multinomial logistic regression to investigate the association between the three outcomes and the predictors at baseline (rather than time-varying).

We also used multinomial logistic regression to investigate the association between the predictors at baseline and four unique outcomes that had developed over the 20-year follow-up: diabetes only, CVD only, CVD followed by diabetes, and diabetes followed by CVD [11].

### Sensitivity analyses

We conducted sensitivity analyses to check the robustness of our findings. All analyses were rerun using complete case data (i.e., only data from those women who responded to all 8 surveys).

All analyses were performed using SAS (version 9.4, SAS Institute Inc.). All statistical tests were two-sided, and *P* < 0.05 was considered to be statistically significant.

## Results

### Characteristics of participants

Of 13,714 women aged 45–50 years at baseline, 3.3% (*n* = 451) reported a history of any of the three conditions and were excluded. The final analytic sample comprised 11,941 women who provided information at two or more consecutive surveys from 1996 to 2016 (**Fig 1**).

Descriptive statistics for women at baseline, categorized according to the number of conditions developed during follow-up, are shown in **Table 1**. Over a follow-up period of 20 years, women who developed multimorbidity were more likely to be separated, divorced, or widowed; to have difficulty in managing on their available income; to be overweight or obese; to have hypertension; to have low levels of physical activity; to be smokers; and to have other chronic conditions (depression/anxiety, asthma, cancer, and arthritis) at baseline.

**Table 1:** Baseline characteristics of women by number of conditions from diabetes, heart disease, and stroke developed during follow-up (*N* = 11,914).

Number of conditions	0 (*n* = 9,430)	1 (*n* = 2,088)	≥2 (*n* = 423)	*P* value^a^
**Mean age (SD)**	47.5 (1.5)	47.7 (1.5)	47.8 (1.4)	<0.001
**Country of birth**				0.327
Australia	7,215 (76.5)	1,622 (77.7)	316 (74.7)	
Outside Australia	2,215 (23.5)	466 (22.3)	107 (25.3)	
**Marital status**				<0.001
Married/de facto	7,910 (84.2)	1,742 (83.8)	318 (75.7)	
Separated/divorced/widowed	1,187 (12.6)	284 (13.7)	90 (21.4)	
Never married	302 (3.2)	54 (2.6)	12 (2.7)	
**Area of residence**				0.268
Major cities	3,462 (36.7)	725 (34.7)	141 (33.3)	
Inner regions	3,568 (37.8)	841 (40.3)	177 (41.8)	
Outer regions	1,929 (20.5)	414 (19.8)	86 (20.3)	
Remote/very remote	469 (5.0)	108 (5.2)	19 (4.5)	
**Education**				<0.001
University/higher degree	1,456 (15.4)	256 (12.3)	40 (9.5)	
Trade/apprenticeship/diploma	1,919 (20.4)	365 (17.5)	82 (19.4)	
High school certificate	1,623 (17.2)	351 (16.8)	63 (14.9)	
No/low qualifications	4,432 (47.0)	1,116 (53.5)	238 (56.3)	
**Ability to manage on income**				<0.001
Easy/not bad	5,601 (59.6)	1,097 (52.8)	176 (42.0)	
Sometimes difficult	2,610 (27.8)	633 (30.5)	131 (31.3)	
Impossible/difficult always	1,185 (12.6)	348 (16.8)	112 (26.7)	
**BMI**				
Underweight (<18.5 kg/m^2^)	165 (1.8)	26 (1.3)	4 (1.0)	<0.001
Normal weight (18.5–24.9 kg/m^2^)	5,130 (55.6)	722 (35.8)	129 (31.2)	
Overweight (25–29.9 kg/m^2^)	2,611 (28.3)	637 (31.6)	126 (30.5)	
Obese (≥30 kg/m^2^)	1,318 (14.3)	630 (31.3)	154 (37.3)	
**Hypertension**	734 (8.1)	287 (14.4)	72 (18.1)	<0.001
**Physical activity**				<0.001
High (**≥**1,200 MET min/week)	1,640 (17.5)	253 (12.2)	59 (14.0)	
Moderate (600–1,199 MET min/week)	2,485 (26.5)	490 (23.7)	103 (24.4)	
Low (40–599 MET min/week)	2,881 (30.7)	668 (32.2)	121 (28.6)	
Nil/sedentary (0–39 MET min/week)	2,375 (25.3)	661 (31.9)	140 (33.1)	
**Smoking status**				<0.001
Never-smoker	5,077 (55.0)	1,048 (51.6)	178 (43.7)	
Ex-smoker	2,629 (28.5)	601 (29.6)	117 (28.8)	
Current smoker	1,521 (16.5)	383 (18.9)	112 (27.5)	
**Other chronic conditions**				
Depression/anxiety	997 (11.0)	229 (11.5)	63 (15.8)	0.010
COPD	653 (7.2)	136 (6.8)	38 (9.6)	0.154
Asthma	730 (8.0)	187 (9.4)	40 (10.1)	0.067
Cancer	405 (4.5)	101 (5.1)	28 (7.0)	0.036
Arthritis	1,662 (20.4)	528 (28.3)	153 (40.9)	<0.001
Osteoporosis	122 (1.3)	30 (1.5)	9 (2.3)	0.286

Due to missing data, the sum for each characteristic may not equal *n*.

^a^*P* values from ANOVA or chi-squared tests.

Abbreviations: ANOVA, analysis of variance; BMI, body mass index; COPD, chronic obstructive pulmonary disease; MET, metabolic equivalent.

### Longitudinal progression of diabetes, heart disease, stroke, and multimorbidity

**Fig 2:**
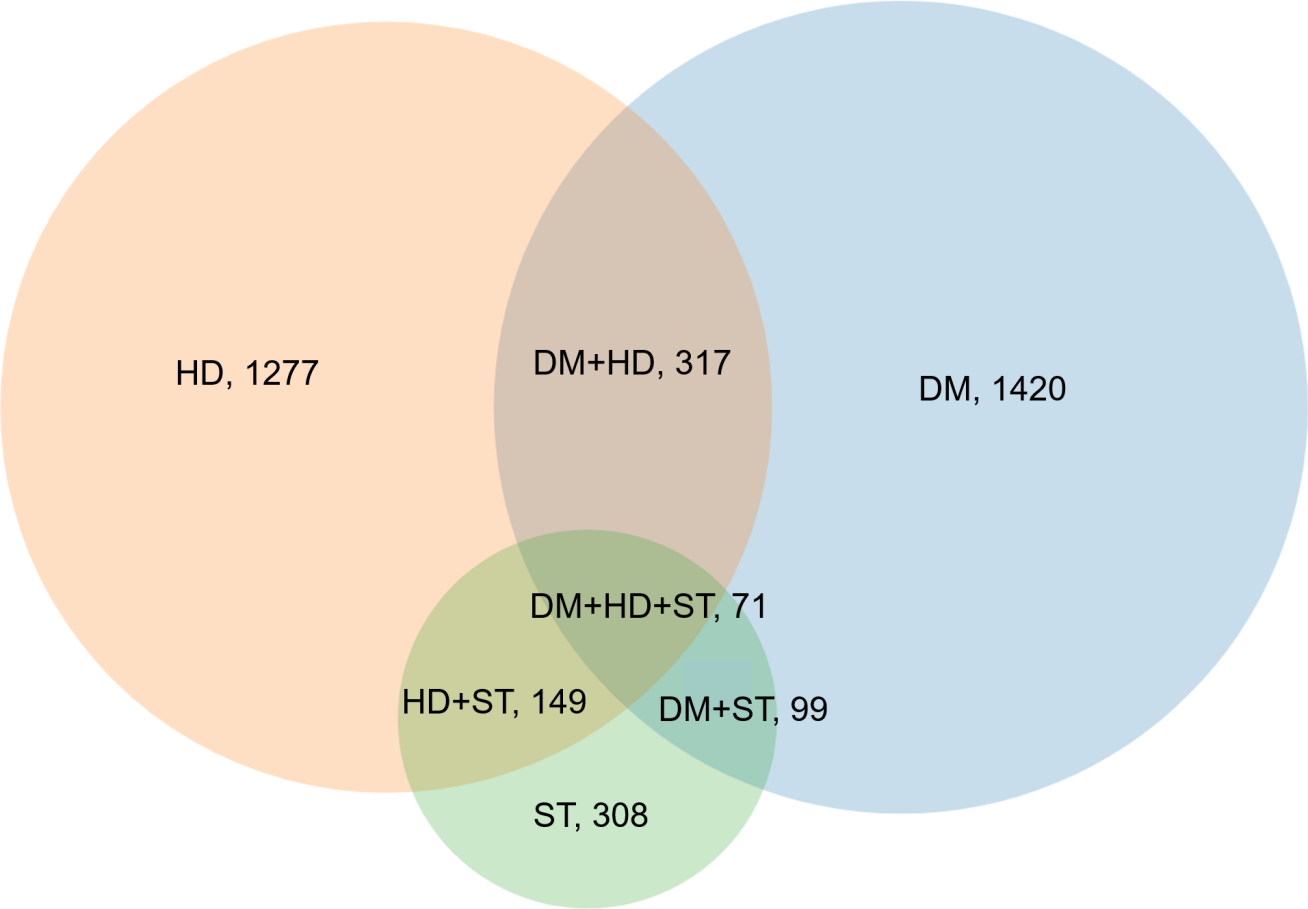
Venn diagram displaying the overlapping combinations of diabetes, heart disease, and stroke in women who developed any condition over the 20 years (*N* = 2,511). DM, diabetes; HD, heart disease; ST, stroke.

During the 20 years of follow-up, 2,511 (18.3%) of the women progressed to at least one condition, of whom 1,420 (56.6%) had diabetes, 1,277 (50.9%) had heart disease, and 308 (12.3%) had stroke; 423 (16.8%) had 2 or 3 of these conditions (**Fig 2**). As expected, there was an increase in the proportion of women with one or more conditions over time, culminating in 19.2% with 1 condition and 3.9% with multimorbidity at Survey 8. Proportions of women with each combination of these conditions at each survey are presented in **S2 Table**.

**Fig 3:**
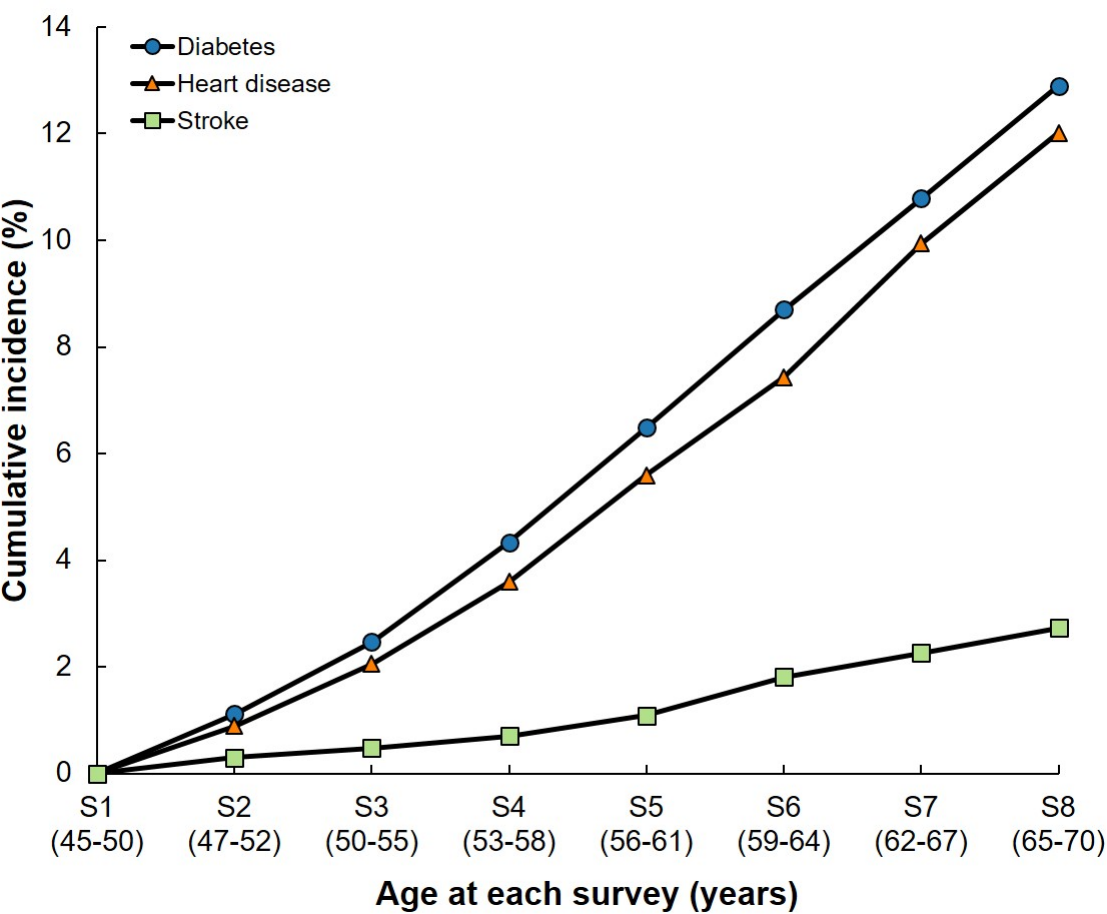
Cumulative incidence of diabetes, heart disease, and stroke in middle-aged Australian women (1996–2016). S, survey number.

**Fig 4:**
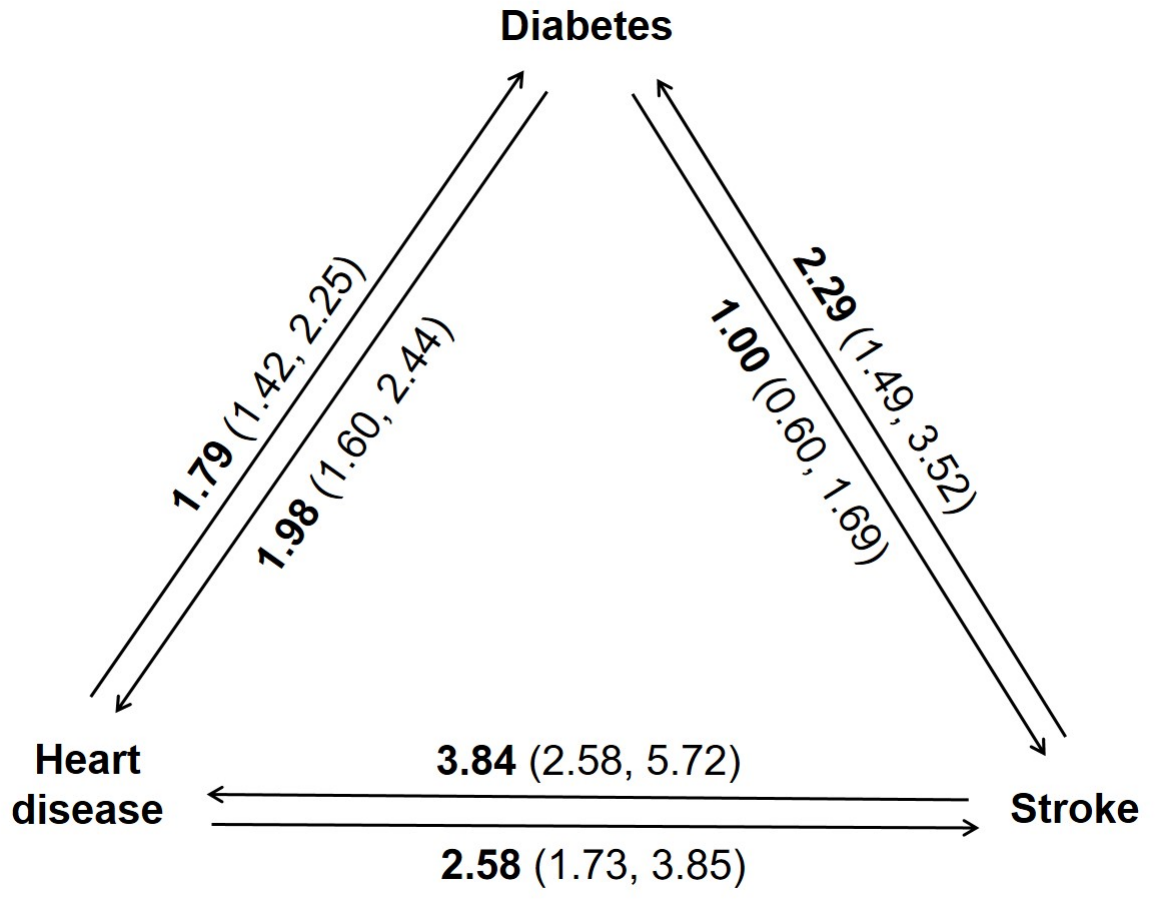
Associations among diabetes, heart disease, and stroke. The ORs and 95% CIs in the figure were estimated using repeated measures logistic regression of existing conditions on the incidence of each of the other two conditions, adjusted for age at baseline and time period. CI, confidence interval; OR, odds ratio.

The cumulative incidence of the three individual conditions is presented in **Fig 3**, with 12.9%, 12.0%, and 2.7% of women progressing to diabetes, heart disease, and stroke by Survey 8, respectively. The three conditions were associated with each other (**Fig 4)**. For example, compared with women without stroke, the OR for progressing to diabetes was 2.29 (95% CI, 1.49–3.52) and to heart disease was 3.84 (95% CI, 2.58–5.72) in women with stroke.

**Fig 5:**
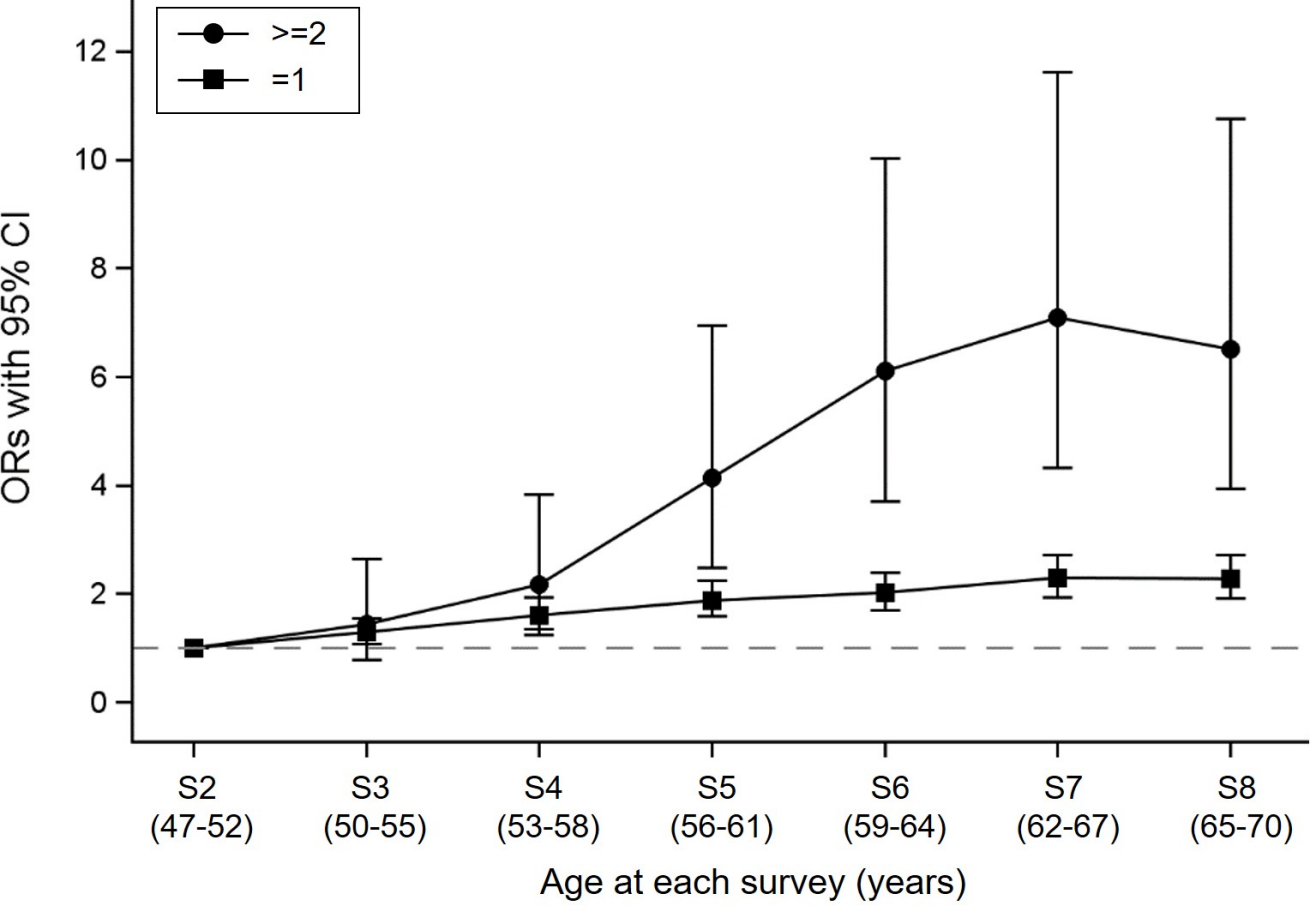
Longitudinal odds of progressing to any one condition and accumulation of multimorbidity. The dashed line indicates the reference group of women who did not develop any new conditions. The ORs were estimated using repeated measures logistic regression, adjusted for age at baseline. The legend “≥2” indicates the transition from none or one to two or three, or from two to three conditions. CI, confidence interval; OR, odds ratio; S, survey number.

The odds for progressing to one condition and accumulation of multimorbidity increased over time, with the accumulation of multimorbidity accelerating between Surveys 4 and 5 (**Fig 5**). Over a 3-year period, the age-adjusted odds of two or more conditions were approximately twice that of developing one new condition, compared to women who did not develop any new conditions. For example, the odds for developing one new condition between Surveys 7 and 8 were 2.29 (95% CI, 1.93–2.72), whereas the odds for developing two or more conditions were 6.51 (95% CI, 3.95–10.75).

**Fig 6:**
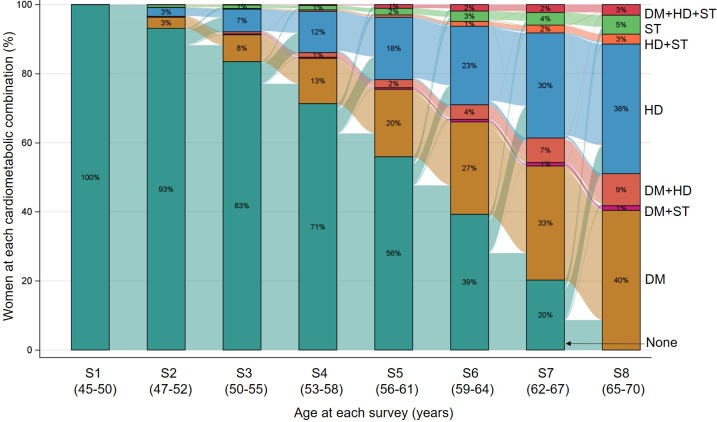
Sankey diagram showing the longitudinal progression and transitions among different combinations of diabetes, heart disease, and stroke in women who developed at least one of these conditions (*N* = 2,511). The bars with different colors show the distribution of different conditions at each survey, and the links between bars show the flow from one condition to another. DM, diabetes; HD, heart disease; S, survey number; ST, stroke.

**Fig 7:**
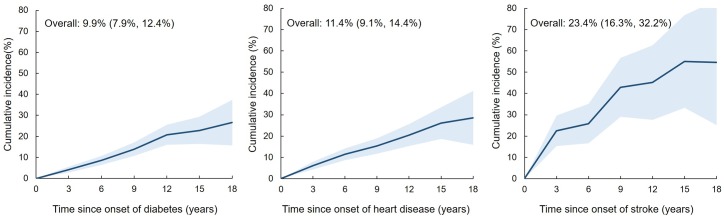
The cumulative incidence of the other two conditions after the first onset of each index condition. Left, diabetes (*N* = 1,193); middle, heart disease (*N* = 996); right, stroke (*N* = 171). Time 0 indicates the first onset of the index condition. Solid lines represent cumulative incidence, with the shaded bands representing 95% CIs. The three figures were plotted using estimates from repeated measures logistic regression, adjusted for age at time 0. CI, confidence interval.

The Sankey diagram in **Fig 6** illustrates the longitudinal progression and transitions among different combinations of diabetes, heart disease, and stroke in women who developed at least one of these conditions. This diagram shows the transitions from “healthy” state (no condition) to any one condition and then to multimorbidity. Over the 20 years of follow-up, the highest rate of progression from one condition to another was observed in women with stroke (**Fig 7**). The overall incidences of multimorbidity after the first onset of diabetes, heart disease, and stroke were 9.9% (95% CI, 7.9%–12.4%), 11.4% (95% CI, 9.1%–14.4%), and 23.4% (95% CI, 16.3%–32.2%), respectively.

### Predictors of accumulation of diabetes, heart disease, and stroke multimorbidity

After controlling for other covariates, being separated, divorced, or widowed; having lower education level; being born in another country; and having difficulties in managing on income were significantly associated with increased odds of accumulating multimorbidity (**Table 2**). A U-shaped relationship between BMI and the incidence of one condition and accumulation of multimorbidity was seen; obesity was associated with 2.6- and 3.0-fold increased odds of one condition and accumulation of multimorbidity (OR = 2.58, 95% CI, 2.28–2.92 and 3.01, 95% CI, 2.21–4.08, respectively). Compared with women without prior hypertension, the OR for progressing to one condition was 1.55 (95% CI, 1.40–1.71) and for accumulation of multimorbidity was 2.19 (95% CI, 1.74–2.75). Being sedentary and current smoking were also associated with increased odds of one condition and accumulation of multimorbidity, as were a number of other chronic conditions (**Table 2**).

**Table 2:** Associations of time-varying sociodemographic and lifestyle factors with 3-year incidence of one condition and accumulation of multimorbidity (*N* = 11,941).

Characteristics	Number of new condition(s)	
	1	≥2^a^
**Age at baseline**	1.07 (1.04–1.11)	1.03 (0.96–1.11)
**Marital status**		
Married/de facto	Ref	Ref
Separated/divorced/widowed	1.03 (0.92–1.17)	1.55 (1.21–1.98)
Never married	1.00 (0.75–1.32)	1.62 (0.93–2.83)
**Area of residence**		
Major cities	Ref	Ref
Inner regions	1.03 (0.93–1.15)	1.23 (0.96–1.58)
Outer regions	0.99 (0.86–1.13)	1.12 (0.82–1.52)
Remote/very remote	1.04 (0.81–1.32)	0.97 (0.52–1.82)
**Education**^**b**^		
University/higher degree	Ref	Ref
Trade/apprenticeship/diploma	1.03 (0.87–1.22)	1.29 (0.86–1.93)
High school certificate	1.18 (0.99–1.40)	1.27 (0.83–1.94)
No qualifications	1.18 (1.02–1.37)	1.45 (1.01–2.10)
**Country of birth**^**b**^		
Australia	Ref	Ref
Outside Australia	1.11 (0.99–1.24)	1.61 (1.26–2.05)
**Ability to manage on income**		
Easy/not bad	Ref	Ref
Sometimes difficult	1.18 (1.05–1.32)	0.97 (0.73–1.28)
Impossible/difficult always	1.46 (1.28–1.66)	1.72 (1.31–2.26)
**BMI**		
Underweight (<18.5 kg/m^2^)	1.39 (0.88–2.19)	1.75 (0.63–4.85)
Normal weight (18.5–24.9 kg/m^2^)	Ref	Ref
Overweight (25–29.9 kg/m^2^)	1.50 (1.32–1.70)	1.77 (1.30–2.43)
Obese (≥30 kg/m^2^)	2.58 (2.28–2.92)	3.01 (2.21–4.08)
**Hypertension**		
No	Ref	Ref
Yes	1.55 (1.40–1.71)	2.19 (1.74–2.75)
**Physical activity**		
High (≥1,200 MET min/week)	Ref	Ref
Moderate (600–1,199 MET min/week)	1.08 (0.94–1.23)	0.93 (0.67–1.28)
Low (40–599 MET min/week)	1.13 (1.00–1.28)	1.06 (0.79–1.41)
Nil/sedentary (0–39 MET min/week)	1.22 (1.07–1.41)	1.38 (1.02–1.86)
**Smoking status**		
Never-smoker	Ref	Ref
Ex-smoker	1.07 (0.96–1.18)	1.22 (0.96–1.55)
Current smoker	1.28 (1.11–1.48)	1.78 (1.31–2.42)
**Other chronic conditions**		
Depression/anxiety	1.25 (1.13–1.39)	1.46 (1.17–1.83)
COPD	1.06 (0.94–1.21)	1.17 (0.90–1.52)
Asthma	1.14 (1.01–1.29)	1.34 (1.04–1.72)
Cancer	1.06 (0.91–1.24)	1.49 (1.11–1.99)
Arthritis	1.20 (1.07–1.35)	1.45 (1.13–1.86)
Osteoporosis	1.10 (0.94–1.29)	1.45 (1.07–1.96)

^a^indicates the transition from none or one to two or three, or from two to three conditions.

^b^not time-varying.

The results (ORs and 95% CI) were estimated using cumulative incidence of multimorbidity (1 or ≥2) at each survey regressed on covariate values at the previous survey, compared with women who developed 0 new conditions. The model was adjusted for all predictors shown in the table.

Abbreviations: BMI, body mass index; CI, confidence interval; COPD, chronic obstructive pulmonary disease; MET, metabolic equivalent; OR, odds ratio.

Similar findings were observed based on multinomial logistic regression of the predictors at baseline (**S3 Table**). However, being sedentary, having depression/anxiety, asthma, cancer, or osteoporosis at baseline were not associated with the incidence of multimorbidity during follow-up using this simpler model.

Similar findings were also observed with the multinomial regression models of the different combinations of these conditions (**S4 Table**). Although obesity was consistently associated with multimorbidity, the odds varied depending on the condition sequence. For example, the OR for diabetes followed by CVD was 1.85 (95% CI, 1.25–2.73), whereas for CVD followed by diabetes it was 2.43 (95% CI, 1.28–4.29).

### Sensitivity analyses

The trends of cumulative incidence of the three individual conditions and the accumulation of multimorbidity were similar when analyses were performed on the complete case data (*N* = 6,718 women), with 12.1%, 11.8%, and 2.9% of women progressing to diabetes, heart disease, and stroke at Survey 8, respectively (**S1 Fig**). The ORs for the incidence of one condition and accumulation of multimorbidity among sociodemographic and lifestyle factors and other chronic conditions were also consistent in complete cases, compared with the main results using all cases (**S5 Table**).

## Discussion

We found that the age-adjusted odds of two or more conditions were approximately twice that of developing one new condition compared to women who did not develop any new conditions, and the onset of stroke is associated with increased risk of progression to heart disease and diabetes. Furthermore, being separated, divorced, or widowed; being born outside Australia; having difficulties with management on income; being overweight or obese; being physically inactive; having hypertension; currently smoking; and having other chronic conditions (i.e, mental disorders, asthma, cancer, osteoporosis, and arthritis) were also associated with this progression.

### Comparison with other studies

There has been an extensive discussion in the literature on the associations of diabetes with heart disease and stroke [5–9,12,13,20,21]. However, the longitudinal progression of these conditions remains unclear. To our knowledge, this is the first study to delineate the progression of diabetes, heart disease, and stroke multimorbidity.

The Emerging Risk Factor Collaboration of 91 cohort studies showed that cardiometabolic multimorbidity (defined as a history of two or three from diabetes, stroke, myocardial infarction) is associated with increased mortality risk (hazard ratios for mortality of only one condition, a combination of any two, and a combination of all three were about 2, 4, and 8, respectively), suggesting that development of cardiometabolic conditions accelerates progression to death [4]. In this study, we focused on the early stages of the disease course to investigate progression to individual conditions and multimorbidity. Our results showed that progression to cardiometabolic conditions accelerates as women age, in particular after their mid-50s (**Fig 5**). The findings of these two studies collectively suggest that interventions that could slow progression to cardiometabolic conditions may have the potential to lead to significant increases in life expectancy.

The relationships among the three conditions are complex. Evidence from systematic reviews suggests that compared with men with diabetes, women with diabetes have a greater risk of coronary heart disease and stroke [8,9]. One population-based cohort study suggested that one-third of patients with heart disease developed new-onset diabetes or impaired fasting glucose during 3.5 years of follow-up [10]. Our study is consistent with these findings. Furthermore, we found that all three conditions were associated with each other, and the onset of stroke may be associated with the progression to other conditions. With self-reported data, a potential explanation for the onset of diabetes following heart disease or stroke is that these women had undiagnosed diabetes (or “prediabetes”) before their heart disease or stroke event. However, statins are recommended for secondary prevention following heart disease or stroke in Australia [22]; hence, heart disease or stroke could have led to statin use, which may have increased the risk of diabetes. Our previous study using data from the 1921–1926 cohort of ALSWH found a 33% increased risk of new-onset diabetes associated with statin exposure [23].

A pooled analysis of individual-level data from 16 cohort studies suggested that overweight and obesity (at baseline) were strongly associated with the incidence of cardiometabolic multimorbidity (at least two from type 2 diabetes, coronary heart disease, and stroke) during a mean follow-up of 10.7 years [11]. Our study is consistent with this study, using multiple follow-up intervals of 3 years and treating BMI as a time-varying variable. However, we found smaller ORs for obesity at baseline on the different combinations of conditions during follow-up. In our study, the ORs associated with obesity varied between 1.10 and 2.43 (95% CI varied between 0.93 and 4.29) depending on the combination of conditions, compared to the previously reported ORs that ranged from 1.5 to 29.8 (95% CI varied between 1.2 and 40.8). Possible explanations for this difference are that the participants in our study were women aged 45–50 at baseline, and they were followed up for 20 years, whereas the previous study included both men and women with a wider age range of 35–105 and a shorter period of follow-up (10.7 years).

The relationship between hypertension and individual diabetes, heart disease, and stroke is well established [24,25], but the results are controversial when considering the sequences of these conditions [24]. We found that women with prior hypertension are more like to progress to multimorbidity. This finding suggests that hypertension might play a major role in the longitudinal progression of the three conditions over time [25]. There have been few studies that have investigated the associations of other chronic conditions with diabetes, heart disease, and stroke multimorbidity [21,26]. A cross-sectional study using data from the UK Biobank found rheumatoid arthritis was associated with the increasing numbers of the three conditions [21], a result consistent with our findings.

### Strengths and limitations

A strength of this study is the large, nationally representative sample and the longitudinal nature of the data, which allowed us to document disease progression over time [27]. Also, as the onset and experience of chronic conditions often occur during midlife [28], the use of a cohort of middle-aged women who were initially healthy reflects the “real-world experience” of disease progression, particularly in the early stages of the course of disease [29]. Furthermore, we used time-varying lifestyle factors and other chronic conditions to capture the dynamic changes of these predictors over time [30]. This may be important, as illustrated by the differing results for the effect of physical activity on the incidence of multimorbidity depending on whether this variable was treated as time-varying or fixed at baseline.

Several methodological limitations of the study should also be noted. First, diabetes, heart disease, stroke, and other chronic conditions are based on self-report, which may have introduced bias in outcome ascertainment. However, the cumulative incidence of the three conditions at the age of 65–70 (Survey 8) in our study population was almost the same as that for women of this age reported in the Australian National Health Survey (2014–2015) [31]. Furthermore, previous studies have validated self-report of various chronic diseases in the ALSWH. For example, the prevalence and bias-adjusted kappa for diabetes, heart disease, stroke, and hypertension from hospital data were 0.93, 0.91, 0.98, and 0.53, respectively [17]. Cancer information included in the study has been validated against Cancer Registry data with 89% sensitivity and 97% specificity [32]. Several other studies have demonstrated the validity of self-reported conditions, BMI, physical activity, and other risk factors [19,33–38].

Second, the small number of cases with more than one condition, potential undiagnosed cases of diabetes, and unknown information on heart disease and stroke type [39,40] might all have led to increased uncertainty and potential underestimation of the odds obtained from the regression models. Third, although we conducted a sensitivity analysis to check the robustness of our findings with complete case data, we did not account for the competing risk of women dying before they could potentially progress to one or more of diabetes, heart disease, and stroke [41]. However, we compared the mortality of all participants and of just the women who developed the three conditions, and the proportions were quite similar and low (4.63% [553/11,941] versus 4.70% [118/2,511], respectively). Fourth, although the attrition rates were low during the 20-year follow-up (**S1 Table**), there is potential for bias in the estimates presented due to attrition or restricting the analysis to complete cases. Fifth, the study sample was women aged 45–50 at baseline, which limits the generalizability of the findings to other groups. However, the study sample is broadly representative of all women born in 1945–1950 in Australia [15]. Finally, the inclusion of information on treatments in the analysis may have influenced the results.

Further studies with a large number of validated cases and for women and men in different age groups are needed to establish the generalizability of these findings and to explore interventions that might slow cardiometabolic progression [27,42].

### Conclusion

Our findings indicate that the odds for accumulation of diabetes, heart disease, and stroke multimorbidity was higher than the odds of progressing to one condition in middle-aged women. Stroke was associated with increased risk of progression to diabetes and heart disease. Social inequality, overweight and obesity, hypertension, physical inactivity, smoking, and having prior chronic conditions (i.e., mental disorders, asthma, cancer, osteoporosis, and arthritis) are all associated with increased risk of accumulation of multimorbidity.

These findings could have significant clinical and public health implications for the treatment and prevention of diabetes, heart disease, and stroke multimorbidity. Delineation of the disease progression may assist in the evaluation of risk for additional conditions during clinical practice. The identified risk factors from this study are appropriate targets for reducing the risk of multimorbidity.

## Supporting information

S1 Checklist STROBE statement—Checklist of items that should be included in reports of cohort studies. (PDF)

S1 Fig Cumulative incidence of diabetes, heart disease, and stroke in middle-aged Australian women in complete cases (*N* = 6,718). (PDF)

S1 Table Retention rates of included participants during follow-up. (PDF)

S2 Table Proportions of women with each combination of diabetes, heart disease, and stroke at each survey in middle-aged Australian women (1996–2016). (PDF)

S3 Table Associations of sociodemographic and lifestyle factors at baseline with 20-year incidence of one condition and multimorbidity (ORs and 95% CIs, *N* = 11,941). CI, confidence interval; OR, odds ratio.
(PDF)

S4 Table Multinomial logistic regression analysis of the associations of sociodemographic and lifestyle factors at baseline with different combinations of conditions during 20-year follow-up (ORs and 95% CIs, *N* = 11,941). CI, confidence interval; OR, odds ratio.
(PDF)

S5 Table Associations of sociodemographic and lifestyle factors with incidence of one condition and multimorbidity in complete cases (*N* = 6,718). (PDF)

S1 Text Proposed analysis plan and modifications following comments from editors and reviewers. (PDF)

## Abbreviations

ALSWH: Australian Longitudinal Study on Women’s Health
ANOVA: analysis of variance
BMI: body mass index
CI: confidence interval
COPD: chronic obstructive pulmonary disease
CVD: cardiovascular disease
ICD-10-AM: International Statistical Classification of Diseases-10^th^ Revision
MET: metabolic equivalent
OR: odds ratio
STROBE: Strengthening the Reporting of Observational Studies in Epidemiology
