# Supplementary material for: Progression of diabetes, heart disease, and stroke multimorbidity in middle-aged women: A 20-year cohort study
Source: PLoS Med. 2018 Mar 13;15(3):e1002516. doi: 10.1371/journal.pmed.1002516 (PMC5849280; doi:10.1371/journal.pmed.1002516)
Supplement: S1 Checklist — (PDF) [file pmed.1002516.s001.pdf]

S1 Checklist. STROBE Statement—Checklist of items that should be included in reports of *cohort studies*

	Item No	Recommendation	In this paper
**Title and abstract**	1	(*a*) Indicate the study's design with a commonly used term in the title or the abstract	Done (a **20-year cohort study** in Title).
		(*b*) Provide in the abstract an informative and balanced summary of what was done and what was found	Done (in the Methods and Findings section).
**Introduction**			
Background/rationale	2	Explain the scientific background and rationale for the investigation being reported	Done (paragraph 1 and 2 in Introduction).
Objectives	3	State specific objectives, including any prespecified hypotheses	Done (paragraph 3 and 4 in Introduction)
**Methods**			
Study design	4	Present key elements of study design early in the paper	Done (paragraph 1 in Methods), for example, '**The Australian Longitudinal Study on Women's Health (ALSWH) is an ongoing population-based cohort study that aims to investigate factors associated with health and well-being over time. '**
Setting	5	Describe the setting, locations, and relevant dates, including periods of recruitment, exposure, follow-up, and data collection	These are presented in the Methods section, for example, '**The women were randomly selected from the national database of Health Insurance Commission, the universal health insurance scheme which includes all citizens and permanent residents of Australia.**' and **'Self-administered questionnaires were sent to the women every three years (apart from a two-year interval between the first and second surveys) until 2016.'**
Participants	6	(*a*) Give the eligibility criteria, and the sources and methods of selection of participants. Describe methods of follow-up	Done (Participants section in Method). We also included a flow diagram to show the selection of participants (**Fig 1.**)
		(*b*) For matched studies, give matching criteria and number of exposed and	NA (the current study is not a matched study).

		unexposed	
Variables	7	Clearly define all outcomes, exposures, predictors, potential confounders, and effect modifiers. Give diagnostic criteria, if applicable	These are presented in the Outcomes and Covariates section in Methods.<br>The Outcomes included the definition and validation of the three-cardiometabolic conditions (**At each survey, women were asked 'Have you ever been told by a doctor that you have: diabetes/…stroke/…heart disease (defined as the presence of angina, or myocardial infarction) over the past 3 years?'. The three self-reported conditions were validated with hospital discharge data in a sub set (New South Wales, Australia) of this cohort with following ICD-10-AM diagnosis codes: diabetes mellitus (E10, E11, E13, and E14), ischemic heart diseases (I20-I25) and stroke (I60-I64) [17]. The prevalence and bias adjusted Kappa for the three conditions were 0.93, 0.91 and 0.98 respectively'** and **'The incidence of each of the conditions was based on the first report of that condition. Accumulation of multimorbidity was based on the first report of two or three of these conditions and the progression from two to three conditions.**<br>The Covariates included the list of covariates and definition of some variables (e.g., BMI, physical activity).
Data sources/ measurement	8*	For each variable of interest, give sources of data and details of methods of assessment (measurement). Describe comparability of assessment methods if there is more than one group	These are presented in the Outcomes and Covariates section in Methods. The detailed sources of each covariates were included in the **S1 Text (Sources of predictors)**.
Bias	9	Describe any efforts to address potential sources of bias	We described related information in various places. For example, 'The women were randomly selected

			from the national database of Health Insurance Commission, the universal health insurance scheme which includes all citizens and permanent residents of Australia.' and 'Women who participated in at least two consecutive surveys with relevant information on exposures and outcomes of interest were included in the analysis'. We investigated through sensitivity analysis the potential impact of missing data by conducting a complete case analysis. Further we considered the possibility of bias due to self-reports of cardiometabolic conditions by considering previous validation studies, comparison with population estimates of cumulative incidence, and speculation on how unreliability of self-reports may have impacted on the odds ratio estimates.
Study size	10	Explain how the study size was arrived at	We also included a flow diagram to show the changing of study size over time (**Fig 1.**) and an **attrition rates and reasons for dropout at each survey** table (**S1 Table**).
Quantitative variables	11	Explain how quantitative variables were handled in the analyses. If applicable, describe which groupings were chosen and why	These are presented in the Statistical analysis, for example:<br>'**BMI was calculated as weight in kilograms divided by height in meters squared and categorised as underweight (<18.5 kg/m2), normal weight (18.5-24.9 kg/m2), overweight (25-29.9 kg/m2), or obese (≥ 30 kg/m2) according to the World Health Organisation classification [19]. Physical activity was categorized as sedentary (0-39 metabolic equivalent [MET] min/week), low (40-599 MET min/week), moderate (600-1199 MET min/week) and high (>= 1200 MET min/week) [20].**'<br>'**Baseline characteristics were described by the**

			**number of cardiometabolic conditions developed during the 20-year follow up (Survey 2 to Survey 8).**',<br>'**We calculated ORs with 95% CIs for the association between the three outcomes at each survey and the risk factors (included time-varying covariates except for education and country of birth) at the previous survey; women with 0 conditions were the reference group. For example, the cumulative incidence of 1, or >=2 conditions from Survey 6 to Survey 7 were modelled using predictors from Survey 6.**'
Statistical methods	12	(*a*) Describe all statistical methods, including those used to control for confounding	These are presented in the Statistical analysis.
		(*b*) Describe any methods used to examine subgroups and interactions	These are presented in the Statistical analysis.
		(*c*) Explain how missing data were addressed	All analyses were re-run using complete cases only (i.e. those women who responded to all eight surveys).
		(*d*) If applicable, explain how loss to follow-up was addressed	As mentioned above, we reported on complete case in sensitivity analysis. Also, missing data for other variables are described for simple descriptive analyses in Table 1's legend.<br>We also mentioned this in the Strengths and Limitations section (**Fourth, although the attrition rates were low during the 20-year follow up (S1 Table), there is potential for bias in the estimates presented due to attrition or restricting the analysis to complete cases.**)
		(*e*) Describe any sensitivity analyses	Done (Sensitivity analyses section in Statistical analysis).
**Results**			

Participants	13*	(a) Report numbers of individuals at each stage of study—eg numbers potentially eligible, examined for eligibility, confirmed eligible, included in the study, completing follow-up, and analysed	Done (Fig. 1 and S1 Table).
		(b) Give reasons for non-participation at each stage	Done (S1 Table).
		(c) Consider use of a flow diagram	Done (Fig. 1).
Descriptive data	14*	(a) Give characteristics of study participants (eg demographic, clinical, social) and information on exposures and potential confounders	Characteristics of study participants are presented in Characteristics of participants section and Table 1 of the Results.
		(b) Indicate number of participants with missing data for each variable of interest	Done (Table 1 and S2 Table).
		(c) Summarise follow-up time (eg, average and total amount)	Follow up was for 20 years or until death (N=597) or until dropout (N=3301).
Outcome data	15*	Report numbers of outcome events or summary measures over time	These are presented in the 'Longitudinal progression of cardiometabolic conditions and multimorbidity' section of Results.
Main results	16	(*a*) Give unadjusted estimates and, if applicable, confounder-adjusted estimates and their precision (eg, 95% confidence interval). Make clear which confounders were adjusted for and why they were included	Fig 2-7 and Table 2.
		(*b*) Report category boundaries when continuous variables were categorized	Two variables:<br>**Body Mass Index**<br>Underweight (<18.5 kg/m2)<br>Normal weight (18.5-24.9 kg/m2)<br>Overweight (25-29.9 kg/m2)<br>Obesity (≥ 30 kg/m2)<br><br>**Physical activity**<br>High (>= 1200 MET min/week)<br>Moderate (600-1199 MET min/week)<br>Low (40-599 MET min/week)<br>Nil/sedentary (0-39 MET min/week)

		(*c*) If relevant, consider translating estimates of relative risk into absolute risk for a meaningful time period	Fig 7. included the cumulative incidence of cardiometabolic multimorbidity after the first onset of each index condition.
Other analyses	17	Report other analyses done—eg analyses of subgroups and interactions, and sensitivity analyses	Sensitivity analyses section of Results.
**Discussion**			
Key results	18	Summarise key results with reference to study objectives	Done (paragraph 2 in the Discussion).
Limitations	19	Discuss limitations of the study, taking into account sources of potential bias or imprecision. Discuss both direction and magnitude of any potential bias	Strengths and Limitations section of Discussion.
Interpretation	20	Give a cautious overall interpretation of results considering objectives, limitations, multiplicity of analyses, results from similar studies, and other relevant evidence	Comparison with other studies section of Discussion.
Generalisability	21	Discuss the generalisability (external validity) of the study results	These are presented in the last paragraph of Discussion (**Fifth, the study sample was women aged 45-50 at baseline, which limits the generalizability of the findings to other groups. However, the study sample is broadly representative of all women born in 1945 to 1950 in Australia [15].**) and in the Conclusion.
**Other information**			
Funding	22	Give the source of funding and the role of the funders for the present study and, if applicable, for the original study on which the present article is based	Done.

*Give information separately for exposed and unexposed groups.

Note: An Explanation and Elaboration article discusses each checklist item and gives methodological background and published examples of transparent reporting. The STROBE checklist is best used in conjunction with this article (freely available on the Web sites of PLoS Medicine at http://www.plosmedicine.org/, Annals of Internal Medicine at http://www.annals.org/, and Epidemiology at http://www.epidem.com/). Information on the STROBE Initiative is available at http://www.strobe-statement.org.
