# Supplementary material for: Progression of diabetes, heart disease, and stroke multimorbidity in middle-aged women: A 20-year cohort study
Source: PLoS Med. 2018 Mar 13;15(3):e1002516. doi: 10.1371/journal.pmed.1002516 (PMC5849280; doi:10.1371/journal.pmed.1002516)
Supplement: S1 Fig — (PDF) [file pmed.1002516.s002.pdf]

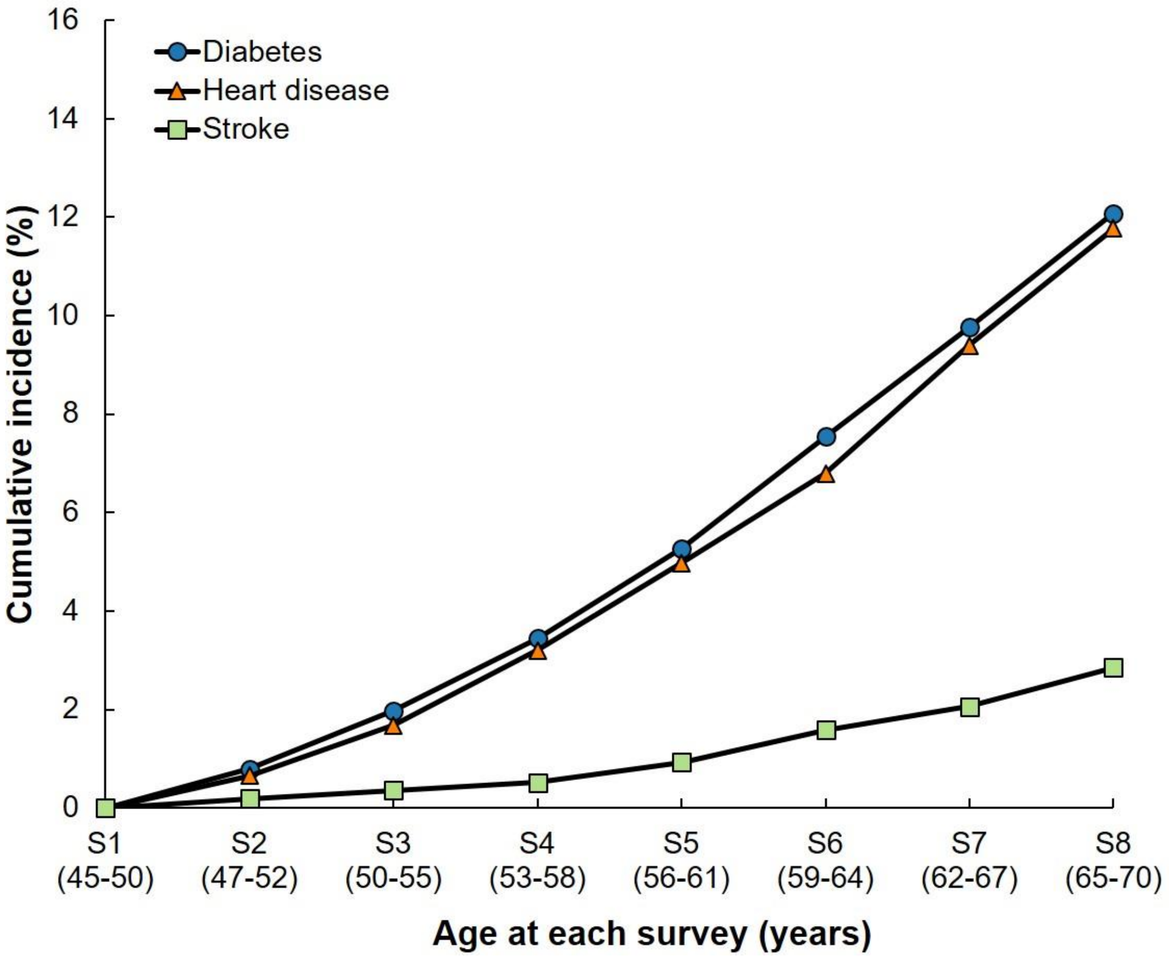

S1 Fig. Cumulative incidence of diabetes, heart disease and stroke in middle-aged Australian women in complete cases (N=6718).
