# Supplementary material for: Progression of diabetes, heart disease, and stroke multimorbidity in middle-aged women: A 20-year cohort study
Source: PLoS Med. 2018 Mar 13;15(3):e1002516. doi: 10.1371/journal.pmed.1002516 (PMC5849280; doi:10.1371/journal.pmed.1002516)
Supplement: S1 Table — (PDF) [file pmed.1002516.s003.pdf]

S1 Table. Retention rates of included participants during follow-up.

	Survey 1	Survey 2	Survey 3	Survey 4	Survey 5	Survey 6	Survey 7	Survey 8
Year	1996	1998	2001	2004	2007	2010	2013	2016
Age	45-50	47-52	50-55	53-58	56-61	59-64	62-67	65-70
Deceased [a]	0	-	76	150	241	352	474	553
Non-respondents	0	456	1353	1540	1685	2086	2742	3164
Respondents (%) [b]	11941	11485 (96.2)	10512 (88.0)	10251 (85.8)	10015 (83.9)	9503 (79.6)	8725 (73.1)	8224 (68.9)

[a] Numbers for deceased are cumulative over surveys.

[b] %, Percentage of respondents at each survey who provided data on at least two consecutive surveys.
