# Supplementary material for: Progression of diabetes, heart disease, and stroke multimorbidity in middle-aged women: A 20-year cohort study
Source: PLoS Med. 2018 Mar 13;15(3):e1002516. doi: 10.1371/journal.pmed.1002516 (PMC5849280; doi:10.1371/journal.pmed.1002516)
Supplement: S2 Table — (PDF) [file pmed.1002516.s004.pdf]

S2 Table. Proportions of women with each combination of diabetes, heart disease and stroke at each survey in middle-aged Australian women (1996-2016).

Cardiometabolic outcome [a]	Survey 1 (n=11941)	Survey 2 (n=11485)	Survey 3 (n=10512)	Survey 4 (n=10251)	Survey 5 (n=10015)	Survey 6 (n=9503)	Survey 7 (n=8725)	Survey 8 (n=8224)
None	11941 (100)	11242 (97.88)	10034 (95.37)	9466 (92.34)	8871 (88.58)	8066 (84.88)	7059 (80.91)	6330 (76.97)
DM	-	114 (0.99)	231 (2.20)	377 (3.68)	533 (5.32)	645 (6.79)	704 (8.07)	793 (9.64)
HD	-	84 (0.73)	178 (1.69)	295 (2.88)	431 (4.30)	508 (5.35)	604 (6.92)	690 (8.39)
ST	-	26 (0.23)	30 (0.29)	42 (0.41)	47 (0.47)	75 (0.79)	79 (0.91)	94 (1.14)
DM+HD	-	10 (0.09)	19 (0.18)	43 (0.42)	72 (0.72)	114 (1.20)	162 (1.86)	187 (2.27)
DM+ST	-	1 (0.01)	4 (0.04)	7 (0.07)	11 (0.11)	18 (0.19)	19 (0.22)	22 (0.27)
HD+ST	-	6 (0.05)	13 (0.12)	15 (0.15)	25 (0.25)	35 (0.37)	52 (0.60)	52 (0.63)
DM+HD+ST	-	2 (0.02)	3 (0.03)	6 (0.06)	25 (0.25)	42 (0.44)	46 (0.53)	56 (0.68)

DM, diabetes; HD, heart disease; ST, stroke.
