# Supplementary material for: Progression of diabetes, heart disease, and stroke multimorbidity in middle-aged women: A 20-year cohort study
Source: PLoS Med. 2018 Mar 13;15(3):e1002516. doi: 10.1371/journal.pmed.1002516 (PMC5849280; doi:10.1371/journal.pmed.1002516)
Supplement: S3 Table — CI, confidence interval; OR, odds ratio. (PDF) [file pmed.1002516.s005.pdf]

S3 Table. Associations of sociodemographic and lifestyle factors at baseline with 20-year incidence of one condition and multimorbidity (ORs and 95% CIs, N=11941).

Characteristics	Number of new condition(s)	
	**1**	**>=2**
**Age at baseline (single year of age)**	1.08 (1.04, 1.12)	1.09 (1.01, 1.18)
**Marital status**		
Married/de facto	Ref	Ref
Separated/divorced/widowed	1.00 (0.84, 1.18)	1.72 (1.30, 2.29)
Never married	0.76 (0.54, 1.07)	0.91 (0.47, 1.76)
**Area of residence**		
Major cities	Ref	Ref
Inner regions	1.02 (0.90, 1.16)	1.18 (0.90, 1.53)
Outer regions	0.91 (0.78, 1.06)	1.06 (0.78, 1.46)
Remote/Very remote	0.96 (0.74, 1.25)	1.07 (0.62, 1.85)
**Education**	1.02 (0.90, 1.16)	1.18 (0.90, 1.53)
University/Higher degree	Ref	Ref
Trade/apprenticeship/diploma	0.99 (0.81, 1.20)	1.55 (1.00, 2.39)
High school certificate	1.09 (0.89, 1.33)	1.44 (0.92, 2.28)
No qualifications	1.11 (0.94, 1.32)	1.48 (0.99, 2.20)
**Country of birth**		
Australia	Ref	Ref
Outside Australia	0.98 (0.86, 1.12)	1.29 (0.99, 1.68)
**Ability to manage on income**		
Easy/not bad	Ref	Ref
Sometime difficult	1.16 (1.02, 1.31)	1.36 (1.04, 1.77)
Impossible/difficult always	1.31 (1.11, 1.54)	2.13 (1.59, 2.87)
**BMI**		
Underweight (<18.5 kg/m^2)	1.34 (0.86, 2.09)	0.77 (0.24, 2.49)
Normal weight (18.5-24.9 kg/m^2)	Ref	Ref
Overweight (25-29.9 kg/m^2)	1.52 (1.33, 1.73)	1.75 (1.32, 2.32)
Obese (≥ 30 kg/m^2)	2.97 (2.58, 3.42)	3.66 (2.76, 4.86)
**Hypertension**		
No	Ref	Ref
Yes	1.64 (1.38, 1.93)	1.94 (1.43, 2.64)
**Physical activity**		
High (>= 1200 MET [a] min/week)	Ref	Ref
Moderate (600-1199 MET min/week)	1.28 (1.06, 1.55)	1.17 (0.81, 1.70)
Low (40-599 MET min/week)	1.49 (1.25, 1.79)	1.03 (0.72, 1.49)
Nil/sedentary (0-39 MET min/week)	1.61 (1.34, 1.94)	1.31 (0.91, 1.88)
**Smoking status**		
Never-smoker	Ref	Ref
Ex-smoker	1.12 (0.98, 1.26)	1.2 (0.92, 1.57)
Current smoker	1.27 (1.09, 1.48)	2.00 (1.51, 2.65)
**Other chronic conditions**		
No other condition	Ref	Ref

Depression/anxiety	0.91 (0.76, 1.08)	1.18 (0.86, 1.62)
COPD	0.89 (0.71, 1.11)	1.13 (0.75, 1.69)
Asthma	1.13 (0.92, 1.38)	0.97 (0.65, 1.44)
Cancer	1.14 (0.88, 1.47)	1.46 (0.93, 2.29)
Arthritis	1.31 (1.16, 1.49)	2.00 (1.58, 2.53)
Osteoporosis	0.84 (0.53, 1.34)	1.15 (0.55, 2.40)

The results (ORs and 95% CI) were estimated using the incidence of multimorbidity (0, 1, or >=2) during the 20-year follow up and associated with predictors at baseline, compared with women who developed 0 condition. The model was adjusted for all predictors shown in the table.

[a] MET, metabolic equivalent.
