# Supplementary material for: Progression of diabetes, heart disease, and stroke multimorbidity in middle-aged women: A 20-year cohort study
Source: PLoS Med. 2018 Mar 13;15(3):e1002516. doi: 10.1371/journal.pmed.1002516 (PMC5849280; doi:10.1371/journal.pmed.1002516)
Supplement: S4 Table — CI, confidence interval; OR, odds ratio. (PDF) [file pmed.1002516.s006.pdf]

S4 Table. Multinomial logistic regression analysis of the associations of sociodemographic and lifestyle factors at baseline with different combinations of conditions during 20-year follow up (ORs and 95% CIs, N=11941).

Characteristics	Condition combinations			
	DM only	CVD only	DM followed by CVD [b]	CVD followed by DM [b]
**Age at baseline**	1.07 (1.02, 1.13)	1.09 (1.04, 1.15)	1.05 (0.95, 1.16)	1.17 (1.01, 1.35)
**Marital status**				
Married/de facto	Ref	Ref	Ref	Ref
Separated/divorced/widowed	0.85 (0.66, 1.08)	1.28 (1.05, 1.56)	1.26 (0.84, 1.91)	1.61 (0.95, 2.71)
Never married	0.63 (0.37, 1.05)	0.87 (0.57, 1.32)	0.95 (0.41, 2.21)	0.87 (0.26, 2.89)
**Area of residence**				
Major cities	Ref	Ref	Ref	Ref
Inner regions	0.96 (0.80, 1.14)	1.11 (0.94, 1.30)	1.17 (0.82, 1.68)	0.94 (0.57, 1.56)
Outer regions	0.94 (0.77, 1.16)	0.88 (0.72, 1.08)	1.12 (0.74, 1.70)	0.96 (0.53, 1.73)
Remote/Very remote	0.94 (0.66, 1.34)	1.03 (0.74, 1.45)	0.60 (0.24, 1.53)	1.50 (0.64, 3.51)
**Education**				
University/Higher degree	Ref	Ref	Ref	Ref
Trade/apprenticeship/diploma	1.09 (0.82, 1.45)	0.99 (0.78, 1.25)	1.31 (0.73, 2.33)	1.42 (0.64, 3.18)
High school certificate	1.40 (1.06, 1.87)	0.91 (0.71, 1.18)	1.38 (0.76, 2.50)	1.43 (0.62, 3.29)
No qualifications	1.41 (1.10, 1.81)	0.99 (0.80, 1.21)	1.28 (0.76, 2.16)	1.28 (0.61, 2.68)
**Country of birth**				
Australia	Ref	Ref	Ref	Ref
Outside Australia	1.20 (1.00, 1.43)	0.86 (0.72, 1.03)	1.09 (0.75, 1.58)	1.42 (0.87, 2.32)
**Ability to manage on income**				
Easy/not bad	Ref	Ref	Ref	Ref
Sometime difficult	1.23 (1.03, 1.45)	1.09 (0.93, 1.28)	1.45 (1.03, 2.06)	1.53 (0.92, 2.54)
Impossible/difficult always	1.41 (1.13, 1.75)	1.28 (1.04, 1.57)	2.04 (1.36, 3.04)	2.61 (1.52, 4.49)
**Body mass index**				
Underweight (<18.5 kg/m^2)	0.97 (0.42, 2.23)	1.47 (0.90, 2.40)	1.11 (0.26, 4.64)	-
Normal weight (18.5-24.9 kg/m^2)	Ref	Ref	Ref	Ref
Overweight (25-29.9 kg/m^2)	2.37 (1.95, 2.87)	1.10 (0.93, 1.30)	1.85 (1.25, 2.73)	2.43 (1.38, 4.29)

Obese (≥ 30 kg/m^2)	5.99 (4.94, 7.26)	1.42 (1.17, 1.72)	4.57 (3.13, 6.67)	5.83 (3.36, 10.14)
**Hypertension**				
No	Ref	Ref	Ref	Ref
Yes	1.82 (1.47, 2.24)	1.51 (1.22, 1.89)	1.67 (1.10, 2.55)	2.21 (1.31, 3.75)
**Physical activity**				
High (>= 1200 MET [a] min/week)	Ref	Ref	Ref	Ref
Moderate (600-1199 MET min/week)	1.15 (0.88, 1.51)	1.42 (1.12, 1.8)	1.04 (0.65, 1.68)	1.10 (0.52, 2.32)
Low (40-599 MET min/week)	1.45 (1.12, 1.86)	1.52 (1.2, 1.91)	0.85 (0.53, 1.37)	1.39 (0.69, 2.80)
Nil/sedentary (0-39 MET min/week)	1.65 (1.28, 2.13)	1.62 (1.28, 2.05)	1.03 (0.65, 1.65)	1.34 (0.66, 2.73)
**Smoking status**				
Never-smoker	Ref	Ref	Ref	Ref
Ex-smoker	1.14 (0.97, 1.36)	1.12 (0.95, 1.31)	0.97 (0.68, 1.40)	1.51 (0.91, 2.49)
Current smoker	1.25 (1.02, 1.55)	1.31 (1.08, 1.59)	1.84 (1.26, 2.67)	2.70 (1.60, 4.56)
**Other chronic conditions**				
No other condition	Ref	Ref	Ref	Ref
Depression/anxiety	0.89 (0.70, 1.14)	0.94 (0.76, 1.18)	1.04 (0.67, 1.63)	1.40 (0.81, 2.42)
COPD	0.89 (0.66, 1.21)	0.90 (0.68, 1.19)	1.21 (0.70, 2.08)	1.00 (0.49, 2.07)
Asthma	1.04 (0.73, 1.50)	1.27 (0.93, 1.73)	1.19 (0.61, 2.31)	1.61 (0.75, 3.46)
Cancer	1.19 (0.91, 1.56)	1.06 (0.82, 1.37)	0.67 (0.37, 1.23)	1.64 (0.89, 3.03)
Arthritis	1.07 (0.90, 1.28)	1.65 (1.41, 1.92)	1.85 (1.35, 2.54)	1.93 (1.25, 2.99)
Osteoporosis	0.77 (0.40, 1.49)	0.88 (0.50, 1.57)	1.68 (0.70, 4.03)	0.76 (0.17, 3.30)

The results (ORs and 95% CI) were estimated using different combinations of conditions during the 20-year follow up and associated with predictors at baseline, compared with women developed 0 condition. The model was adjusted for all predictors shown in the table.

[a] MET, metabolic equivalent.

[b] CVD followed by diabetes refers to women with heart disease or stroke who subsequently developed comorbid diabetes. Diabetes followed by CVD refers to women with diabetes who subsequently developed comorbid heart disease or stroke.
