# Supplementary material for: Progression of diabetes, heart disease, and stroke multimorbidity in middle-aged women: A 20-year cohort study
Source: PLoS Med. 2018 Mar 13;15(3):e1002516. doi: 10.1371/journal.pmed.1002516 (PMC5849280; doi:10.1371/journal.pmed.1002516)
Supplement: S5 Table — (PDF) [file pmed.1002516.s007.pdf]

S5 Table. Associations of time-varying sociodemographic and lifestyle factors with 3-year incidence of one condition and accumulation of multimorbidity in complete cases (N=6718).

Characteristics	Number of new condition(s)	
	**1**	**>=2 [a]**
**Age at baseline**	1.07 (1.04, 1.11)	1.04 (0.96, 1.11)
**Marital status**		
Married/de facto	Ref	Ref
Separated/divorced/widowed	0.89 (0.76, 1.04)	1.43 (1.05, 1.95)
Never married	0.95 (0.67, 1.34)	1.47 (0.74, 2.94)
**Area of residence**		
Major cities	Ref	Ref
Inner regions	1.05 (0.92, 1.19)	1.35 (0.99, 1.83)
Outer regions	0.98 (0.83, 1.16)	1.16 (0.80, 1.70)
Remote/Very remote	1.05 (0.78, 1.43)	1.15 (0.55, 2.42)
**Education [b]**		
University/Higher degree	Ref	Ref
Trade/apprenticeship/diploma	1.00 (0.82, 1.21)	1.29 (0.80, 2.07)
High school certificate	1.09 (0.88, 1.33)	1.23 (0.74, 2.04)
No qualifications	1.10 (0.92, 1.30)	1.33 (0.86, 2.05)
**Country of birth [b]**		
Australia	Ref	Ref
Outside Australia	0.99 (0.86, 1.15)	1.64 (1.22, 2.21)
**Ability to manage on income**		
Easy/not bad	Ref	Ref
Sometime difficult	1.12 (0.98, 1.28)	0.93 (0.67, 1.29)
Impossible/difficult always	1.55 (1.31, 1.83)	1.73 (1.23, 2.45)
**Body mass index**		
Underweight (<18.5 kg/m^2)	1.14 (0.62, 2.10)	0.67 (0.09, 4.92)
Normal weight (18.5-24.9 kg/m^2)	Ref	Ref
Overweight (25-29.9 kg/m^2)	1.43 (1.23, 1.67)	1.47 (0.98, 2.19)
Obese (≥ 30 kg/m^2)	2.41 (2.07, 2.81)	3.37 (2.32, 4.89)
**Hypertension**		
No	Ref	Ref
Yes	1.14 (1.02, 1.28)	2.35 (1.80, 3.06)
**Physical activity**		
High (>= 1200 MET min/week)	Ref	Ref
Moderate (600-1199 MET min/week)	1.08 (0.92, 1.27)	0.71 (0.47, 1.10)
Low (40-599 MET min/week)	1.14 (0.98, 1.33)	1.19 (0.85, 1.67)
Nil/sedentary (0-39 MET min/week)	1.17 (0.99, 1.39)	1.47 (1.02, 2.11)
**Smoking status**		
Never-smoker	Ref	Ref
Ex-smoker	1.13 (1.00, 1.28)	1.22 (0.91, 1.63)
Current smoker	1.38 (1.14, 1.67)	1.86 (1.24, 2.79)
**Other chronic conditions**		
Depression/anxiety	1.33 (1.18, 1.50)	1.62 (1.26, 2.08)
COPD	1.05 (0.90, 1.22)	1.05 (0.78, 1.41)
Asthma	1.17 (1.01, 1.35)	1.33 (1.00, 1.76)

Cancer	0.93 (0.77, 1.13)	1.21 (0.86, 1.70)
Arthritis	1.22 (1.07, 1.40)	1.37 (1.04, 1.80)
Osteoporosis	0.99 (0.82, 1.21)	1.43 (1.02, 2.01)

The results (ORs and 95% CI) were estimated using cumulative incidence of multimorbidity (0, 1, or >=2) at each survey regressed on time-varying covariates at the previous survey. The model was adjusted for all predictors shown in the table.

[a] indicates the transition from none or one to two or three, or from two to three conditions.
[b] not time varying.
