# Supplementary material for: Progression of diabetes, heart disease, and stroke multimorbidity in middle-aged women: A 20-year cohort study
Source: PLoS Med. 2018 Mar 13;15(3):e1002516. doi: 10.1371/journal.pmed.1002516 (PMC5849280; doi:10.1371/journal.pmed.1002516)
Supplement: S1 Text — (PDF) [file pmed.1002516.s008.pdf]

S1 Text. Proposed analysis plan and modifications following comments from editors and reviewers

Research time: July 21 to September 15, 2017.
Research objectives:

- To examine the longitudinal progression of the three conditions (diabetes, heart disease, and stroke) over time;
- To estimate the role of demographic and lifestyle factors in the accumulation of three conditions and multimorbidity.

Participants:

1946-51 cohort of Australian Longitudinal Study on Women's Health (ALSWH).

Outcomes:

- The incidence of three conditions:
  - Diabetes (Type 1 and 2)
  - Heart disease (angina, or myocardial infarction)
  - Stroke
- The cumulative incidence of three disease states: 0 condition, any 1 condition, any combination of 2 or 3 conditions.

Eight patterns of disease progression between adjacent surveys and the definition of cumulative incidence of conditions.

	Survey N	Survey (N+1)	Disease development
	Disease history	Number of new condition	
1	0	0	0 to 0
2	0	1	0 to 1
3	0	2 or 3	0 to 2 or 3
4	1	0	1 stable
5	1	1	1 to 2
6	1	2 or 3	1 to 2 or 3
7	2 or 3	0	2 or 3 stable
8	2	1	2 to 3

Predictors: Self-reported sociodemographic factors, and lifestyle factors (see below table).

Sources of predictors

Covariate	Definition	Source
**Sociodemographic Factors**		
**Country of birth**		Baseline
Australia		
Other	Including United Kingdom, Italy, Greece, New Zealand, Vietnam, and others	
**Marital status**		Each survey
Married/de facto	de facto (opposite sex, same sex)	
Separated/divorced/widowed		
Never married	Single	
**Area of residence**	Using ARIA+ score to define area of residence [a]	Each survey
Major cities	0-0.20	
Inner regions	>0.20-2.40	
Outer regions	>2.40-5.92	
Remote/Very remote	Remote Australia >5.92-10.53<br>Very remote Australia>10.53	
**Education**		Baseline
University/higher degree	University degree, Higher university degree (e.g. Grad Dip, Masters, PhD)	
Trade/apprenticeship/higher	Trade/apprenticeship (e.g. Hairdresser, Chef), Certificate/diploma (e.g. Child Care, Technician)	
Higher school certificate	Higher school or leaving certificate (or equivalent)	
No or low qualifications	No formal qualifications, School or intermediate certificate (or equivalent)	
**Ability to manage on income**		Each survey

Easy/not bad		
Sometimes difficult		
Impossible/difficult always		
**Lifestyle Factors**		
**BMI**	WHO classification	Each survey
Underweight	<18.5 kg/m^2	
Normal weight	18.5-24.9 kg/m^2	
Overweight	25-29.9 kg/m^2	
Obese	≥ 30 kg/m^2	
**Physical activity**	Using MET min/week to define physical activity [b]	Each survey
High	≥ 1200 MET min/week	
Moderate	600-1199 MET min/week	
Low	40-599 MET min/week	
Nil/sedentary	0-39 MET min/week	
**Smoking status**		Each survey
Never-smoker	Have never smoked	
Ex-smoker	Used to smoke	
Current smoker	Smoke occasionally or regularly	

[a] ARIA+ (Accessibility/Remoteness Index of Australia) is Australia's most authoritative geographic measure of remoteness based on distances to major population centres (1).

[b] A physical activity score in metabolic equivalent (MET) minutes per week was derived using the following formula: MET min/week = (walking minutes * 3.5 METs) + (moderate acativity minutes * 4.0 METs) + (vigorous activity minutes * 7.5 METs) (2)

Statistical analyses:

- Descriptive analyses
  - the baseline characteristics of participants
  - the cumulative incidence of every single condition and different multimorbidity combinations at each survey
- Modelling
  - the relationships among the three conditions
  - the associations of predictors and the cumulative incidence of conditions (0, 1, or >=2)

(more statistical analyses were included in the Statistical analyses section of the manuscript)

Additional analyses:

- We conducted sensitivity analyses to check the robustness of our findings with complete cases.

Modification based on the comments from editors and reviewers:

Following the suggestion of reviewers, we also fitted a multinomial logistic regression model to investigate the association between the predictors at baseline and four unique outcomes that had developed over the 20-year follow up: diabetes only, cardiovascular disease (CVD, i.e., heart disease or stroke or both) only, CVD followed by diabetes, and diabetes followed by CVD. CVD followed by diabetes refers to women with heart disease or stroke who subsequently developed comorbid diabetes. Diabetes followed by CVD refers to women with diabetes who subsequently developed comorbid heart disease or stroke.

References

1. The Department of Health 2011;Pageshttp://www.health.gov.au/internet/publications/publishing.nsf/Content/ARIA-Review-Report-2011~ARIA-Review-Report-2011-2~ARIA-Review-Report-2011-2-2-3 on June, 22 2017.
2. Brown WJ, Burton NW, Marshall AL, Miller YD**.** Reliability and validity of a modified self-administered version of the Active Australia physical activity survey in a sample of mid-age women. Aust N Z J Public Health. 2008;32(6):535-41.
